# Association between elevated serum bilirubin levels with preserved lung function under conditions of exposure to air pollution

**DOI:** 10.1186/s12890-021-01488-5

**Published:** 2021-04-13

**Authors:** Udi Shapira, Rafael Y. Brezinski, Ori Rogowski, David Zeltser, Shlomo Berliner, Itzhak Shapira, Shani Shenhar-Tsarfaty, Elizabeth Fireman

**Affiliations:** 1 Departments of Internal Medicine “C”, “D” & “E” and Institute for Special Medical Examinations (MALRAM), Tel-Aviv Sourasky Medical Center, Affiliated to the Sackler Faculty of Medicine, Tel-Aviv University, Tel-Aviv, Israel; 2 Laboratory of Pulmonary and Allergic Diseases, Tel-Aviv Sourasky Medical Center, Affiliated to the Sackler Faculty of Medicine, Tel-Aviv University, 6 Weizman Street, 6423906 Tel-Aviv, Israel

**Keywords:** Normal population, Bilirubin, Air pollution, NOx, CO

## Abstract

**Background:**

High serum bilirubin levels have been shown to be associated with an improved pulmonary function test results. Their potential ability to similarly benefit pulmonary function in an environment of polluted air has not been tested. We retrospectively analyzed data of 15,605 apparently healthy individuals in order to evaluate the effect of serum bilirubin levels on forced expiratory volume in 1 s (FEV1).

**Methods:**

Individuals attended the Tel-Aviv Medical Center Inflammatory Survey for a routine annual health check between February, 2002 and June, 2009 and were divided into low, medium and high serum bilirubin levels. Their FEV1 results were compared under various levels of air pollution. Air pollution and weather data were obtained from air pollution monitoring stations of the Israeli Ministry of Environmental Protection.

**Results:**

The elevated serum bilirubin concentrations on FEV1 were evaluated under moderate and high pollution levels FEV1 and were significantly higher in participants with high blood bilirubin levels compared to medium or low levels (*p* < 0.001 and *p* = 0.018, respectively). Participants with high levels of bilirubin had preserved FEV1 under exposure to high and medium pollution levels of both Nitrogen Oxide (NOx) and Carbon Monoxide (CO) pollutants (*p* = 0.003 and *p* = 0.022, respectively). The multivariate regression analysis revealed that the influence of bilirubin under conditions of air pollution remained significant even after adjustment for FEV1 confounders, but the interaction was not significant.

**Conclusions:**

Elevated serum bilirubin concentrations are associated with preserved lung function in healthy individuals in Israel exposed to high levels of air pollution.

## Background

Chronic respiratory diseases are among the leading causes of morbidity and mortality worldwide [1]. Short- and long-term exposures to elevated air pollution levels lead to deterioration of lung function in individuals of all ages [2–4]. In adults it has been shown that exposure to air pollution cause a decline in pulmonary function by generating oxidative stress in the lung tissue [5, 6]. Bilirubin is the end product of heme breakdown. The majority of bilirubin originates from degradation of erythrocyte hemoglobin in the reticuloendothelial system, while the remaining ~ 20% comes from inefficient erythropoiesis in bone marrow and other heme proteins degradation [7, 8]. Bilirubin was shown to exhibit anti-oxidative and anti-inflammatory properties in vitro and in vivo [9]. In a Swiss study on Air Pollution and Lung Disease in adults (SAPALDIA), increased concentrations of serum bilirubin were associated with improved parameters of forced expiratory volume in 1 s (FEV1) and forced vital capacity (FVC) ratio (FEV1/FVC) and forced expiratory flow at 25–75% of FVC (FEF25–75%), after adjusting for the effects of sex, age, education, height, and weight and tobacco smoke exposure [10]. One longitudinal study demonstrated a significant negative relationship between baseline bilirubin levels, incidence of coronary heart disease (CHD), and mortality from cardiovascular disease (CVD) [11]. Another study showed inverse association between total bilirubin level and CVD risk, independent of other risk factors [12]. The inclusion of total bilirubin in the standard established risk factors panel, however, provided no significant improvement in CVD risk prediction. A large prospective Korean study revealed that a higher basal bilirubin level within the normal range was associated with a low risk of lung cancer. Smoking and low bilirubin levels have been cumulatively associated with a higher risk of lung cancer [13]. Moreover, bilirubin concentrations were negatively associated with the history, duration, and intensity of smoking, suggesting a possible role of inhaled tobacco in the pathogenesis of lung function deterioration related to oxidative stress [14].

To the best of our knowledge, there are no published data on any correlations between bilirubin levels and pulmonary function test results under conditions of air pollution. In the present study, we assessed this correlation and the interaction between functional parameters, levels of bilirubin and air pollution in a large cohort of apparently healthy Israeli individuals who participated in an annual health survey.

## Methods

### Description of study population

**Fig. 1:**
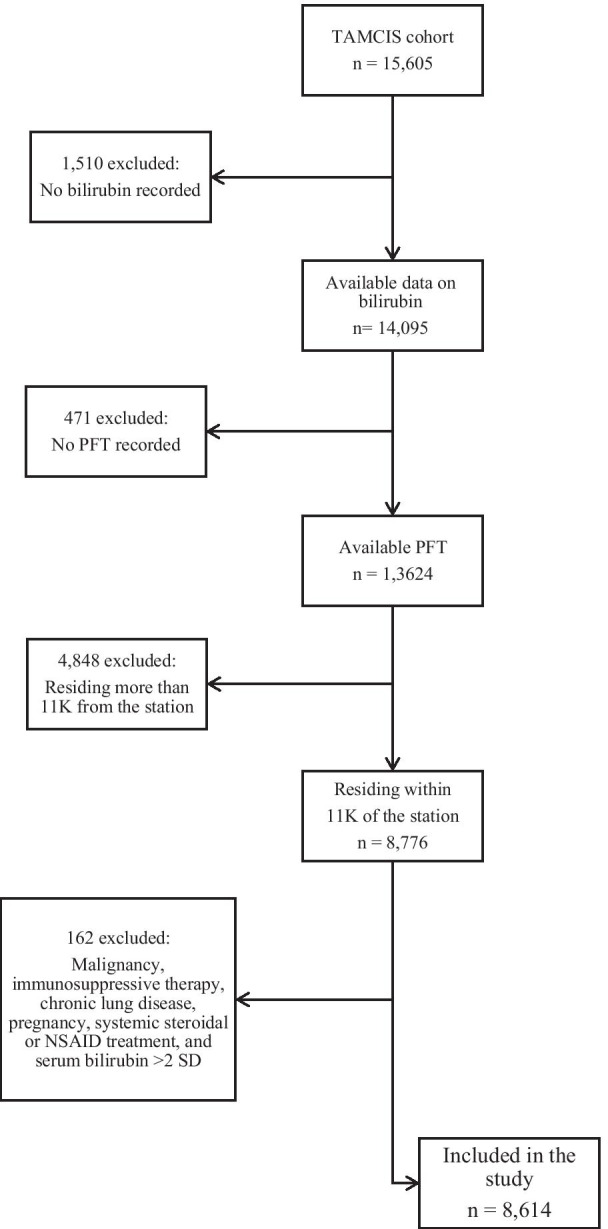
Study population flow chart

The study population included 15,605 individuals who attended the Tel-Aviv Medical Center Inflammatory Survey (TAMCIS) for a routine annual health check between February, 2002 and June, 2009 as previously described [5]. We excluded subjects for whom there were no bilirubin measurements (*n* = 1510), or no complete PFT results (*n* = 471), and those who lived within a radius > 11 km from an air pollution monitoring station (*n* = 4848). We also excluded 162 participants due to a malignancy history, immunosuppressive therapy, chronic lung disease, pregnancy, systemic steroidal or nonsteroidal treatment (except for aspirin at a dose of 325 mg/day), and serum bilirubin concentrations > 2 standard deviations (± 1.48 mg/dL). The final study cohort was comprised of 8614 individuals (Fig. 1).

### Spirometry measurements

The PFT consisted of spirometry with a KoKo spirometer (Ferraris Respiratory, Louisville, Colorado, USA), according to the American Thoracic Society quality criteria [15]. The results are expressed as percentage of predictive values (age, height, and sex), and all tests were performed during the early hours of the day. Three to six trials were performed for each subject. All error-free trials were recorded only after there had been two consecutive reproducible trials. System calibrations were carried out daily. All PFTs were performed by a single highly trained technician.

### Air pollution and weather data

Air pollution and weather data were obtained from air pollution monitoring stations of the Israeli Ministry of Environmental Protection. These stations are located at roof height in representative areas, which are not located inside neighborhoods with excessive sources of toxic emission, such as industrial plants or heavy traffic. All distances of these stations from the subject’s residence were supplied by the Survey of Israel. Temperature and relative humidity were measured every 30 min by the same air pollution monitoring station. The air monitoring stations continuously measure several major pollutants, specifically, carbon monoxide (CO), nitrogen oxide (NOx, generic nitric oxide), and nitrogen dioxide (NO_2_). The monitoring process is fully automated and approved by the U.S. Environmental Protection Agency. Stations undergo daily automated calibration and produce annual quality control and assurance test results. In addition, nitrogen dioxide is measured by a chemiluminescence analyzer (Model 42C, Thermo Environmental Instruments), ozone by a UV photometric O_3_ analyzer (Model 49C, Thermo Environmental Instruments), and carbon monoxide by a gas filter correlation CO analyzer (Model 48C, Thermo Environmental Instruments).

The exclusion of subjects living outside an 11-km radius from an air pollution monitoring station was based on earlier studies on the effect of environmental air pollution on PFT parameters [5, 16]. The levels of selected major pollutants were averaged over the 7 days prior to the participant’s visit to our medical center.

### Statistical analysis

All continuous variables are displayed as means (standard deviation, SD) for normally distributed variables, or median (interquartile range, IQR) for variables with abnormal distribution, and were compared by a Student’s t-test and by Mann–Whitney U-test, respectively. Categorical variables are displayed as numbers (%) of patients within each group, and they were compared by a chi-squared test. The cohort was divided into three groups according to low, moderate, and high bilirubin and pollutants levels. We used one-way ANOVA and the Tukey post-hoc analysis to evaluate the differences within these groups. The contribution of bilirubin levels to changes in FEV1 values under different levels of NOx and CO was evaluated using a logistic regression model. FEV1 was defined as the dependent variable, and the model was adjusted for bilirubin levels, age, body mass index (BMI), sex, smoking status (current/past/never), and diabetes diagnosis (yes/no). All analyses were conducted using the SPSS Statistics 22.0 statistical package (IBM Corporation, Armonk, NY, USA).

## Results

The study population consisted of 4646 (61.2%) men and 2641 (38.8%) women, with a mean age of 44.84 (± 11.27) years. Among them were 1338 (17.8%) current smokers. Individuals with lower FEV1 values (< 80% of predictive values) were more likely to be men, older, and have a higher BMI. The study population’s characteristics are presented in Table 1.

**Table 1:** Demographic and clinical parameters of the study population

*n* (%)	Total	FEV1 ≥ 80%	FEV1 < 80%	*p *value
	7590	7170 (94.5)	420 (5.5)	
Age, year (SD)	44.84 (11.27)	44.65 (11.22)	48 (11.66)	< 0.001*
Sex, male, *n* (%)	4646 (61.2)	4360 (60.8)	286 (68.1)	0.003*
BMI, kg/m^2^ (SD)	26.35 (4.32)	26.26 (4.25)	27.78 (5.2)	< 0.001*
Current smoking,* n* (%)	1338 (17.6)	1225 (17.1)	113 (26.9)	< 0.001*
Past smoking, *n* (%)	1882 (24.8)	1762 (24.6)	120 (28.6)	0.065
Never smoked, *n* (%)	4236 (55.8)	4059 (56.6)	177 (41.1)	< 0.001*
Bilirubin, mg/dL (SD)	0.72 (0.5)	0.72 (0.24)	0.69 (0.22)	0.077
D7 CO (SD)	0.81 (0.28)	0.8 (0.28)	0.87 (0.28)	0.001*
D7 NOx (SD)	33.4 (24.54)	33.25 (24.26)	35.75 (28.8)	0.098
FVC (SD)	101.97 (21.64)	103.27 (21.43)	79.77 (9.8)	< 0.001*
FEV1 (SD)	99.55 (13.6)	101.15 (12.14)	72.31 (6.35)	< 0.001*
FEV1/FVC (SD)	102.17 (8.02)	102.59 (7.6)	95.11 (10.67)	< 0.001*

*SD* standard deviation, *BMI* body mass index, *D7* seven days, *CO* carbon monoxide, *NOx* nitrogen oxide, *FVC* forced vital capacity percent of predictive values, *FEV1* forced expiratory volume in 1 s percent of predictive values. Results are given as mean (*SD* standard deviation)

*p < 0.05 is considered as significant

As expected, current smokers had lower FEV1 and FEV1/FVC values compared to past smokers and participants who never smoked. Additionally, women demonstrated higher FEV1, FVC, and FEV1/FVC values compared to men (Table 2).

**Table 2:** The effect of smoking and sex on pulmonary function test

	Smoking			Sex	
	Smokers	Past smoking	Never smoked	Male	Female
FEV1	97.53 **(14.01)	99.66 (13.92)	(13.2) 100.2^b^	98.72 (13.41)	100.87** (13.78)
FVC	101.88 (30.84)	101.67 (14.21)	102.19 (21.0)	100.13 (24.95)	104.84** ((14.50
FEV1/FVC	100.67 (8.33)**	102.21 (8.08)	102.62 (7.82)	102.0 ((8.12	102.44* ((7.85

*FVC* forced vital capacity, *FEV1* forced expiratory volume in 1 s. Results are given as mean percent of predictive values (standard deviation)

Smoking effect was examined with ANOVA and post-hoc. Sex effect was examined by *t* test

*p value < 0.05; **p value < 0.01

**Fig. 2:**
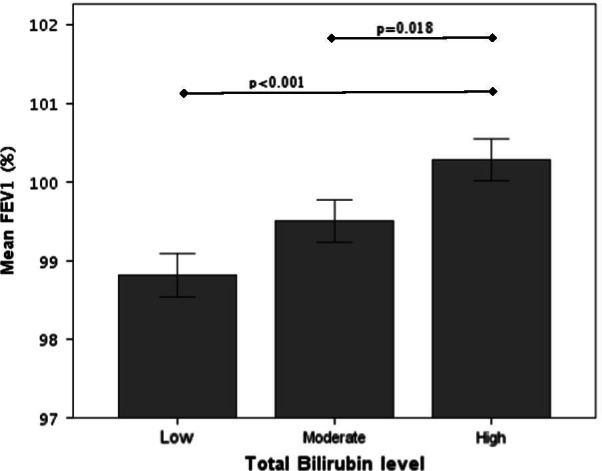
Assessment of FEV1 according to different levels of serum bilirubin. *FEV1* forced expiratory volume in 1 s. Y axis—mean level of FEV1 percent of predictive values. X axis—bilirubin level (mg/dL) divided according to low, moderate, and high serum bilirubin; 0.1–0.59 mg/dL, 0.6–0.8 mg/dL and 0.81–1.47 mg/dL, respectively

To evaluate the effect of total serum bilirubin levels on FEV1, we divided the cohort into low, medium, and high serum bilirubin levels. The results showed that FEV1, but not FVC or FEV1/FVC, was significantly higher in participants with high blood bilirubin compared to low or medium levels (*p* < 0.001 and *p* = 0.018, respectively) (Fig. 2).

**Fig. 3:**
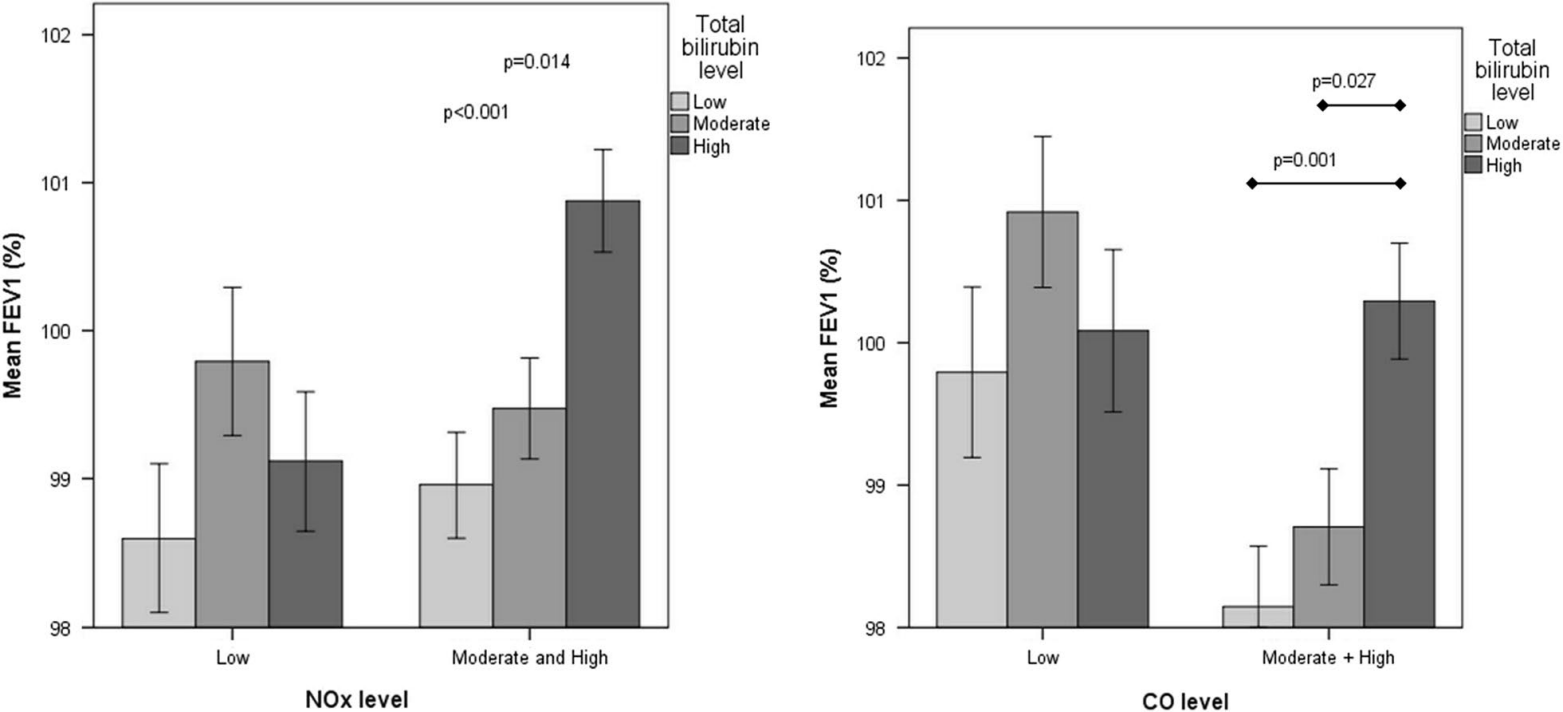
**a** Assessment of FEV1 according to different levels of serum bilirubin under different NOx levels. *FEV1* forced expiratory volume in 1 s, *Nox* nitrogen oxide. Y axis—mean level of FEV1 percent of predictive values. X axis—NOx level (low, medium and high; 0.4–14.0 ppm; 14.1–22.1 ppm and 22.2–33.5 ppm, respectively). Bilirubin level (mg/dL) divided according to low, moderate, and high serum bilirubin; 0.1–0.59 mg/dL, 0.6–0.8 mg/dL and 0.81–1.47 mg/dL, respectively. **b** Assessment of FEV1 according to different levels of serum bilirubin under different CO levels. *FEV1* forced expiratory volume in 1 s, *CO* carbon monoxide. Y axis—mean level of FEV1 percent of predictive values. X axis—CO level (low 0.1–0.7 ppm; medium and high 8–0.9 ppm and 1–1.2 ppm). Bilirubin level (mg/dL) divided according to low, moderate, and high serum bilirubin; 0.1–0.59 mg/dL, 0.6–0.8 mg/dL and 0.81–1.47 mg/dL, respectively

**Fig. 4:**
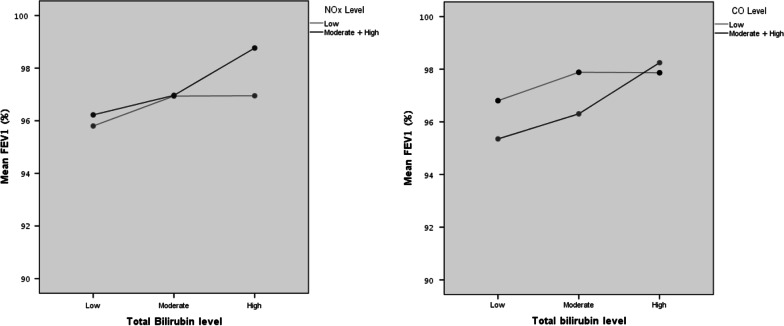
**a** Estimated mean levels of FEV1 in low versus moderate and high levels of bilirubin to NOx adjusted for age, sex, BMI, diabetes, and smoking. *FEV1* forced expiratory volume in 1 s, *Nox* nitrogen oxide, *BMI* body mass index. Y axis—mean level of FEV1 percent of predictive values. X axis—bilirubin level (mg/dL) divided according to low, moderate, and high serum bilirubin; 0.1–0.59 mg/dL, 0.6–0.8 mg/dL and 0.81–1.47 mg/dL, respectively. Gray line: low NOx level 0.4–14.0 ppm; Black line: 14.1–22.1 ppm and 22.2–33.5 ppm. **b** Estimated mean levels of FEV1 in low versus moderate and high levels of exposure to CO adjusted for age, sex, BMI, diabetes, and smoking. *FEV1* forced expiratory volume in 1 s, *CO* carbon monoxide, *BMI* body mass index. Y axis—mean level of FEV1 percent of predictive values. X axis—bilirubin level (mg/dL) divided according to low, moderate, and high serum bilirubin; 0.1–0.59 mg/dL, 0.6–0.8 mg/dL and 0.81–1.47 mg/dL, respectively. Gray line: low CO level 0.1–0.7 ppm; Black line: 0.8–0.9 ppm and 1–1.2 ppm

Next, we explored the possible effect of bilirubin on FEV1 under conditions of exposure to major air pollutants. Importantly, participants with high levels of bilirubin presented with preserved lung function (FEV1 values) under conditions of exposure to high and medium pollution levels of both NOx and CO pollutants (*p* = 0.003 and *p* = 0.022, respectively, Fig. 3a, b). FEV1, under moderate and high level of NOx with high bilirubin, compared to low bilirubin level and moderate bilirubin level is significantly high, *p* = 0.014, and *p* < 0.001 respectively (Fig. 3a). Under moderate and high level of CO with high bilirubin, compared to low bilirubin level and moderate bilirubin level, FEV1 is also significantly high, *p* = 0.027 and *p* = (Fig. 3b). The apparently protective effect of elevated serum bilirubin concentrations on FEV1 under conditions of moderate and high NOx and CO pollution levels also remained significant after adjusting for age, BMI, sex, smoking status, and diabetes diagnosis (Fig. 4a, b). The interactions between air pollutants (NOx or CO) with bilirubin on PFT results were not significant.

## Discussion

The main finding of this study is the positive association between total serum bilirubin concentrations and lung function in subjects exposed to elevated levels of air pollution. Our relatively large study sample (*n* = 15,605) was comprised of apparently healthy individuals residing in a major metropolitan area, supporting the likelihood that our findings are applicable to other large urban areas worldwide.

Our results of elevated serum bilirubin levels being associated with improved lung function are compatible with the findings in Swiss [10] and South Korean [14] populations that also described positive associations between serum bilirubin levels and lung function in healthy populations. Moreover, there was an association between a lower risk of respiratory disease and all-cause mortality in patients with normal-range bilirubin levels, while relatively higher levels of bilirubin were associated with a lower risk of respiratory diseases [17]. In another study, bilirubin levels were associated with a longer 6-min walk distance and a better quality of life [18].

Bilirubin is known as a potential antioxidant with anti-inflammatory properties. Elevated bilirubin levels have cytoprotective properties, including antioxidant, anti-inflammatory, and antiproliferative effects [19]. These effects seem to be mediated by an elevated heme oxygenase 1 (HO-1) activity which may be moderated by genetic variations or environmental factors. The role of HO-1 in correlation with the environment was shown in some other studies as well. Moreover, environmental cadmium moderates diabetes type 2 through HO-1 activity [20] and the effect of environmental toxins, such as cigarette smoke, silica, and asbestos on different lung diseases seem to be mediated by bilirubin derivate HO-1 [21]. In contrast to all of these studies that correlated bilirubin with the environment or the effect of bilirubin on functional parameters of respiration, we extended these investigations and looked at how bilirubin levels and pulmonary function test findings were correlated to environmental pollutants.

Our novel finding is that the positive association between pulmonary function tests and bilirubin was significantly stronger among participants exposed to elevated levels of major air pollutants, such as CO and NOx, suggesting that high levels of circulating bilirubin may have a protective effect against lung impairment under conditions of exposure to ordinary urban air pollution. This led us to focus our attention upon a possible mechanism of this protection looking at the metabolism of HO-1 and its therapeutic effects. The use of pharmacological agents that augment expression of HO-1 has been recently investigated, and the results showed protection against a variety of oxidative stress and inflammatory conditions [22]. Induction of HO-1 by a triterpenoid protected neurons against ischemic injury [23], and HO-1 was expressed more abundantly in the lesions of synovial tissue from rheumatoid arthritis patients than in those from the other patient groups. Hemin, auranofin, and HO-1-expression vector induced HO-1, and reduced expression of Tumor Necrosis Factor may mediate less inflammation in those patients [23]. Consequently, its metabolites, such CO, biliverdin and bilirubin, could become parts of a therapeutic strategy for the treatment of various inflammatory illnesses [24, 25].

We are aware that this study has several limitations that bear mention, one of which is its retrospective design. We do not have follow-up data on major respiratory or cardiovascular events, thus precluding any prognostic application of our findings. In addition, measurements of air pollution levels were made according to the subjects’ residential areas and did not take into account their places of work. Finally, we did not perform any mechanistic-oriented studies.

## Conclusions

The key finding of this study was that elevated serum bilirubin concentrations are associated with preserved lung function in healthy individuals exposed to elevated levels of air pollution. Bilirubin should be further studied as a surrogate marker for lung function in respiratory diseases in which there are elevated levels of oxidative stress.

## Data Availability

The data that support the findings of this study are available from Prof. Shlomo Berliner but restrictions apply to the availability of these data, which were used under license for the current study, and so are not publicly available. Data are however available from the authors upon reasonable request and with permission of Prof. Shlomo Berliner (Principle investigator).

## Abbreviations

BMI: Body mass index
CHD: Coronary heart disease
CO: Carbon monoxide
CVD: Cardiovascular disease
FEF25–75%: Forced expiratory flow at 25–75% of FVC
FEV1: Forced expiratory volume in 1 s
FVC: Forced vital capacity
HO-1: Heme oxygenase 1
NO_2_: Nitrogen dioxide
NOx: Nitrogen oxide
PFT: Pulmonary function test
TAMCIS: Tel-Aviv Medical Center Inflammatory Survey
